# Dynamic Changes of Selected Signaling Molecules in Ovaries Following Early-Life Exposure to Fumonisin B1 in Wistar Rats in Association With DNA Methylation

**DOI:** 10.33549/physiolres.935499

**Published:** 2025-06-01

**Authors:** Awadh ALHELAISI, Saber NAHDI, Abeer ALHAZMI, Saleh ALWASEL, Abdel Halim HARRATH

**Affiliations:** 1 Department of Zoology, College of Science, King Saud University, Riyadh, Kingdom of Saudi Arabia

**Keywords:** Mycotoxin, Fumonisin B1, Epigenetic, Signaling pathway, Methylation, Ovary

## Abstract

The mycotoxin fumonisin B1 (FB1) poses a significant global problem due to its presence in the food chain. This study aimed to investigate the intergenerational effects of FB1 on epigenetic changes and the corresponding signaling pathways in rat ovaries. Specifically, we examined the expression levels of DNA methyl-transferase (Dnmt3b) and the Pi3kK/Akt/mTOR/Ampk pathway. Virgin Wistar albino female rats were divided into control and FB1 treatment (doses of 20 and 50 mg/kg body weight/day) groups and monitored from day 6 of pregnancy until delivery. Female rats from the first (F1) and second (F2) generations were euthanized at 4 weeks of age, and their ovaries were collected. In addition to histopathological damage, there was a significant dose-dependent increase in Dnmt3b protein in the ovaries of F1 females (*p*=0.0022 and *p*<0.0001, respectively), but not in those of F2 females. Furthermore, overexpression of the *PI3K* gene was observed only in the high-dose FB1 group in both the F1 and F2 generations. In F2, significant gene overexpression of *Akt* was observed in the high-dose FB1 group, while no effect was observed in F1. Both treated groups of F1 females showed significant repression of the *mTOR* gene, whereas no effect was observed in F2 females. In addition, FB1 did not have a significant effect on the expression of the *Ampk* gene in either treatment group in either generation. We conclude that early-life exposure to FB1 may directly impact the ovarian function of female rats by altering methylation patterns and *Pi3k/Akt/mTOR* pathway in first-generation females. However, this effect appears to be recoverable in the second-generation females.

## Introduction

It is widely believed that most diseases arise from a combination of genetic predispositions that increase susceptibility, as well as environmental factors [1]. Thus, exposure to environmental factors during fetal and early postnatal development may increase the risk of developing adult-onset disease later in life [2–5]. Stress, exposure to toxins, and irregular nutrition are some environmental factors that can not only cause the direct effects of early-life exposure on adult-onset disease but can also affect subsequent generations [6–9]. In addition to the traditional induction of genetic mutations, the transgenerational inheritance of epigenetic modifications in the genome offers a second molecular mechanism for the germline transmission of environmentally induced phenotypic change [10,11]. If a pregnant female is considered the F0 generation, then the offspring are the F1 generation, and the germ cells of the growing fetuses will eventually develop into eggs or sperm and form the F2 generation. Parental (F0) exposure to environmental factors directly impacts the first generation and therefore the germ cells that are present in the fetuses that produce the F2 generation [12,13]. The study of mitotically and meiotically heritable modifications in gene function or gene expression without altering the DNA sequence is known as epigenetics [14,15].

Numerous studies have investigated the relationship between exposure to chemicals and environmental factors, specifically endocrine-disrupting chemicals (EDCs), and the impact of this exposure on various organs at the epigenetic level [16,11]. These studies suggest that certain disorders can be inherited due to changes in DNA methylation, histone modification, and noncoding RNAs. For instance, it has been reported that certain adult diseases, such as type II diabetes mellitus, obesity, and metabolic syndromes, are associated with epigenetic mechanisms during the reprogramming period in the embryonic stage [17]. Moreover, a study revealed that exposure to a mixture of pesticides and plastics in utero resulted in an increased incidence of cysts resembling human polycystic ovarian disease (PCO) in female offspring [18]. Interestingly, paternal exposure to phthalates has been linked to poor blastocyst quality in couples seeking fertility treatments, and it has been suggested that this exposure is associated with abnormalities in sperm DNA methylation [19,20]. Therefore, DNA methylation, along with other gonad markers, can be used to assess reproductive aging [21,11].

In our previous study, we investigated the intergenerational effects of fumonisin B1 (FB1) on ovarian structure and function [8]. We found that early exposure to high doses of FB1 led to downregulation of the *Cyp19* and *Esr2* genes in female subjects in the first generation, in conjunction with the downregulation of the autophagic marker Lc3. However, fertility recovery among second-generation female subjects was suggested, as most of the ovarian and autophagic markers recovered to normal levels in both treatment groups. In this study, we hypothesized that DNA methylation may be altered across two generations of female Wistar rats following early exposure to FB1, potentially serving as a mechanism for FB1-mediated ovarian toxicity. To investigate this, we analyzed the gene expression of Dnmt3b using quantitative real-time polymerase chain reaction (qPCR) and assessed protein expression through immunofluorescence in the offspring of both the first and second generations after maternal exposure to FB1. Additionally, we evaluated several key signaling molecules that could be involved in the ovarian-disrupting effects of this mycotoxin in relation to DNA methylation.

## Materials and Methods

### Ethical statement

This study was approved by the Scientific Research Ethics Committee at King Saud University, Riyadh, Saudi Arabia (Reference No: KSU-SE-22-41) and was carried out in accordance with the approved guidelines. All animal experiments followed the ARRIVE guidelines and were carried out in accordance with the National Institutes of Health guide for the care and use of Laboratory animals (NIH Publications No. 8023, revised 1978).

### Study design and sampling

Thirty healthy virgin female Wistar-Albino rats, weighing 200–250 g, were housed in separate cages in a facility that maintained a temperature of 21 °C, fed a standard laboratory chow diet, and had a 12-hour photoperiod. Then, they were housed with males. Gestational day 0 (GD 0) was defined as the day a white vaginal plug appeared on the cage floor, indicating that mating was successful. The three groups of pregnant females received the following treatments (fumonisin was dissolved in distilled water) from GD 6 to GD 21: The first group of females (n=10) was given distilled water by gavage and was labeled the control group. The second group of females (n=10) was given an oral dose of 20 mg/kg FB1. The third group of females (n=10) was given an oral dose of 50 mg/kg FB1. FB1was used by dissolving it in distilled water. The doses of treatment were selected based on previous studies [22,23]. After giving birth, the offspring of the mothers who received FB1 treatment were considered the first generation (F1), and the offspring of the control group were considered the first control generation (CF1). In preparation for euthanasia when the offspring were 4 weeks old (before puberty), a portion of the offspring were placed in a clear plastic box with a carbon dioxide tube attached and a flow rate of 10 l/h for 10 min. The ovaries were extracted quickly, measured, cleaned, and divided into groups according to their origins. Once they reached sexual maturity, the remaining females from F1 and CF1 were mated with males. Offspring from FB1-treated F1 mothers comprised the second generation (F2), while control group offspring were referred to as the second control generation (CF2). Similar to their mothers, the ovaries of the female F2 and CF2 offspring were extracted after euthanasia when they were 4 weeks old, after which they were cleaned and labeled accordingly.

### Histological preparation

Ovarian samples were fixed for 24 h in neutral buffered formalin (NBF, 10 %). The following day, the samples were embedded in paraffin and cut into 5–7 μm thick sections. For adhesion, the sections were collected on a hotplate and transferred to glass slides containing warm (30 °C) water and albumin glycerol fixative. Hematoxylin and eosin was used to stain the sections for histological analysis after the wrinkles were removed [4].

### Immunofluorescence staining and confocal microscopy

Immunostaining was carried out as described in previous studies [24]. The tissue sections were placed on a heated plate (60 °C), deparaffinized with xylene, rehydrated and washed twice with distilled water and three times with 1× phosphate-buffered saline (PBS). The slides were dried after the washing process. The slides were placed in a container at room temperature, and tissue sections were permeabilized using 0.1 % Triton X-100 with 0.1 % sodium citrate and treated with blocking buffer (1 % bovine serum albumin (BSA) in PBS). Using a humid box, the slides were incubated with primary antibody solution (DNMT-3, dilution 1:500, DGpeptidesCo. Ltd., Wuhan, China) for 24 h at 4 °C on a flat surface in the dark. Afterward, the slides were washed four times with 1× PBS and treated with the secondary antibody (FITC, dilution 1:700, Abcam, MA, USA) for 45 min at room temperature (RT) in the dark. Next, the slides were washed with PBS followed by TE buffer before adding Hoechst solution (dilution 1:15000, Hoechst 33342, Life Technologies, MA, USA). Finally, the sections were mounted in 50 % glycerol/TE solution, and the edges were covered with nail polish. For signal quantification, the sections were observed and imaged with a spinning disk confocal microscope (Zeiss). The ZEN 3.1 service (ZEN lite) was used to analyze the signal intensity for protein expression, and GraphPad Prism 9 (GraphPad Software) was used for quantification.

### Analysis of gene expression

An RNeasy Mini Kit (Qiagen, Westburg, The Netherlands) with DNase treatment was used to isolate RNA on columns using an RNase-free DNase kit (Qiagen). The quality and purity of the extracted RNA were measured *via* a Nanodrop spectrophotometer at a 260/280 nm ratio. Using RT-PCR and primer sets from an iScript™ cDNA synthesis kit (Applied Biosystems, Carlsbad, CA) according to the manufacturer’s instructions, cDNA was reverse-transcribed into 0.1 to 0.5 μg of total RNA. Finally, real-time PCR (RT-PCR) was performed using SYBR Green and an Applied Biosystems 7500 Fast RT-PCR system (Carlsbad, CA) with the gene-specific primers shown in Table 1. The relative amount was calculated using the 2^−ΔΔCT^ method and normalized against the reference gene GAPDH for each gene transcript.

### Statistical analysis

GraphPad Prism Version 9 was used to analyze the data. For statistical comparisons, one-way analysis of variance followed by Tukey’s multiple comparison test was used. All values are shown as the mean ± standard deviation (SD). A p<0.05 was considered to indicate statistical significance.

## Results

### Histopathological ovarian disruption after in utero exposure to FB1

**Fig. 1:**
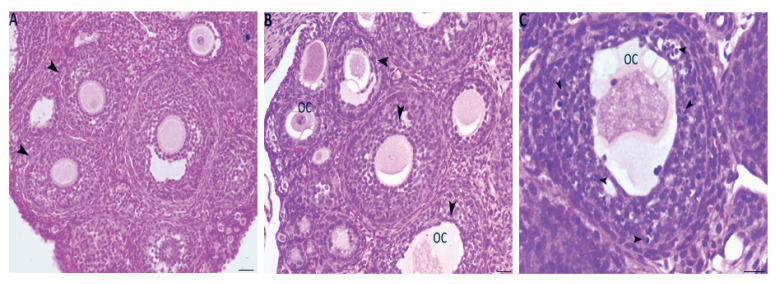
H&E-stained ovarian sections showing histopathological changes in the FB1-treated groups (B and C) compared to the control group (A). (**A**) Ovarian sections from the control group showing a normal structure containing normal growing follicles (arrowhead). (**B**) Different stages of follicles from the FB1-treated group from F1 showing degenerating follicles (arrowhead) and abnormal oocyte structure (oc). (**C**) A follicle from the fumonisin B1-treated group showing granulosa cells with a high number of pyknotic nuclei (arrowheads) and abnormal oocytes with vacuolization.

The ovarian sections of 4-week-old females in the control group (Fig. 1A) showed a normal ovary structure with developing follicles at different stages of maturity. However, compared with those from the control group, the ovarian sections from the low- and high-dose treatment groups showed differences in damage. In particular, the treated females clearly exhibited an abnormal oocyte (oc) structure and degenerating follicles (Fig. 1B). The majority of granulosa cells appeared to have many pyknotic nuclei. The majority of the oocytes were surrounded by a thin, irregular zona pellucida with a vacuolated area (Fig. 1C).

### Global DNA methylation in ovaries of next-generation females is altered by developmental FB1 exposure

**Fig. 2:**
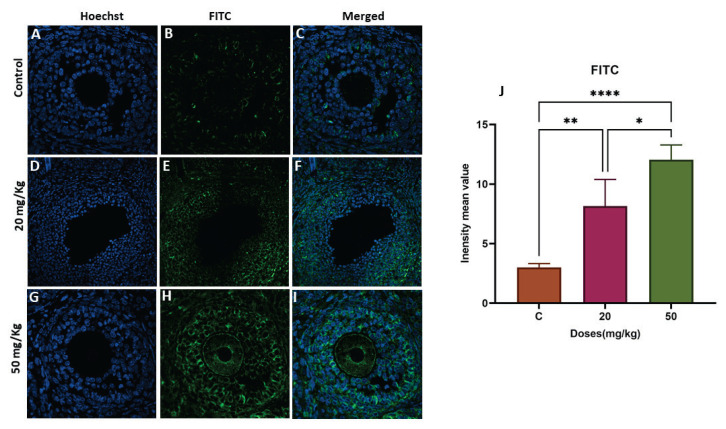
The effect of FB1 exposure on DNMT3b localization in ovarian tissue from F1 females was assessed using immunofluorescence staining in the control (**A–C**), 20 mg/kg FB1-treated (**D–F**), and 50 mg/kg FB1-treated groups (**G–I**). The relative fluorescence intensity (**J**) of DNMT3b was assessed with the ZEN 3.1 service (ZEN lite) and quantified using GraphPad Prism 9 (GraphPad Software 10.1.1). (*) indicates a p<0.05, (**) indicates a p<0.01, and (****) indicates a p<0.0001.

**Fig. 3:**
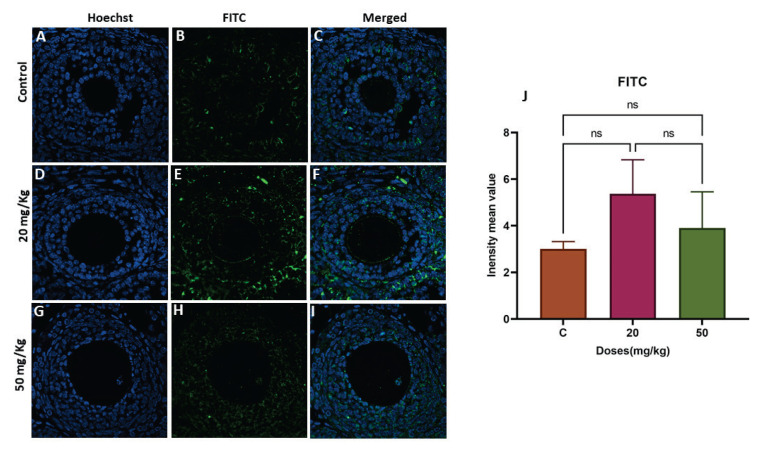
The effect of FB1 exposure on DNMT3b localization in ovarian tissue from F2 females was assessed using immunofluorescence staining in the control (**A–C**), 20 mg/kg FB1-treated (**D–F**), and 50 mg/kg FB1-treated groups (**G–I**). The relative fluorescence intensity (**J**) of DNMT3b was assessed with the ZEN 3.1 service (ZEN lite) and quantified using GraphPad Prism 9 (GraphPad Software 10.1.1). ns: not significant.

To explore whether FB1 induced epigenetic changes across generations, Dnmt3 protein expression were measured using RT-PCR and immunofluorescence staining, respectively. Dnmt3 protein levels were significantly increased in a dose-dependent manner in both first-generation treatment groups (20 and 50 mg/kg) (p=0.0022 and p<0.0001, respectively) (Fig. 2A–J), while no significant effect was detected in either of the second-generation treatment groups (Fig. 3A–J).

### Gene expression of the Pi3k/Akt/mTOR signaling pathway after in utero exposure to FB1

**Fig. 4:**
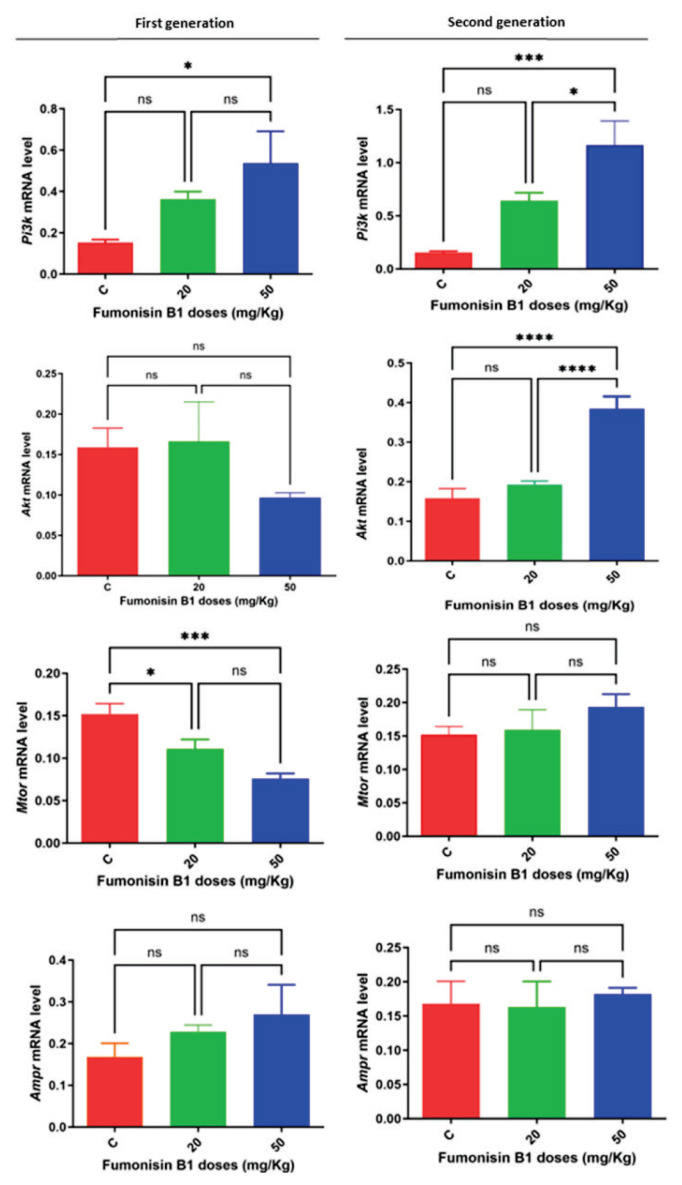
Effects of FB1 on the gene expression of the *Pi3k/Akt/mTOR/Ampk* pathway in rat ovaries from the F1 and F2 generations, as detected by RT-PCR (F–H). (**A**) *Pi3k* mRNA levels in F1; (**B**) *Pi3k* mRNA levels in F2; (**C**) *Akt* mRNA levels in F1; (**D**) *Akt* mRNA levels in F2; (**E**) *mTOR* mRNA levels in F1; (**F**) *mTOR* mRNA levels in F2; (**G**) *Ampk* mRNA levels in F1; (**H**) *Ampk* mRNA levels in F2. The values are presented as the mean ± S.E.M. (*) indicates a p<0.05, (***) indicates a p<0.001, (****) indicates a p<0.0001, and ns: nonsignificant.

To understand the mechanisms by which FB1 affects ovarian cells, we analyzed the Pi3k/Akt/mTOR/Ampk signaling pathway using RT-PCR (Fig. 4). Compared to those of the control group, the expression levels of Pi3k were significantly greater only in the group exposed to a high dose of FB1 in both the first and second generations (p=0.0368 and p=0.0003, respectively) (Fig. 4). Although there were no notable changes in the mRNA levels of the Akt gene in either of the first-generation treatment groups, a significant increase was observed in the second generation of the group exposed to the high dose of FB1 (50 mg/kg) (p<0.0001). The mRNA levels of the mTOR gene were significantly decreased in both treatment groups of the first generation (p=0.03 and p<0.0002, respectively), while no effect was observed in the treatment groups of the second generation. The results demonstrated that FB1 did not have a significant effect on the expression of the Ampk gene in either treatment group or in either generation. Overall, these findings suggest that FB1 has varying effects on the different components of the Pi3k/Akt/mTOR/Ampk signaling pathway.

## Discussion

Our previous study demonstrated that exposure to FB1 during fetal development resulted in reduced fertility due to the dysregulation of various ovarian genes, such as *Cyp19*, *Esr2*, and *Gdf9*. This dysregulation led to an increase in the ovary weight index, particularly with the high-dose treatment (50 mg/kg body weight [b.w.]/day), which is similar to what is observed in polycystic ovary syndrome (PCOS) (Fig. 1). Additionally, there was a decrease in the number of primordial follicles, contributing to an accelerated decline in reproductive capacity associated with aging. Our current study aimed to examine the potential direct association between prenatal exposure to FB1 and DNA methylation in adult rat ovaries. In fact, DNA methylation has been proposed as a method for understanding the mechanisms of ovarian aging [11]. Specifically, we investigated DNA methylation and gene expression in granulosa cells from both young and older subjects to analyze changes in methylation patterns during reproductive aging. In this way, 3397 genes were found to exhibit differential expression between the two groups, including genes known to be associated with ovarian function [25]. In our study, we detected an increase in Dnmt3 protein and gene expression in the first generation of in utero FB1-treated females, showing a direct correlation between in utero exposure to FB1 and global hypermethylation in the adult ovary (Fig. 2). These DNA hypermethylation events, possibly mediated by increased Dnmt3b expression, are linked to changes in gene expression and dysfunction within the adult ovary, leading to decreased fertility. Indeed, epigenetic reprogramming occurs during embryonic development under the effect of many factors, and these epigenetic modifications influence the expression of genes, leading to a new phenotype [26]. Numerous previous studies have demonstrated that alterations in DNA methylation patterns are associated with pathologies in various organs [27]. For example, females exposed to EDCs have been shown to experience pregnancy loss, oocyte aneuploidy, the formation of multioocyte follicles, and disruptions in estrus cyclicity [28,29]. Similarly, exposure to DDT led to an increased prevalence of ovarian polycystic ovarian syndrome and primary ovarian insufficiency [30]. All of these interrelated disorders may originate before birth, probably due to the increasing release of synthetic chemicals into the environment, which may contribute to changes in DNA methylation patterns [27,31].

However, the direct association between prenatal exposure to FB1 and DNA hypermethylation observed in the adult rat ovaries of the F1 females was not observed among the F2 females, as no significant changes in Dnmt3b were found in either of the F2 treatment groups (Fig. 3). This may indicate that the prenatal epigenetic reprogramming that occurred due to FB1 exposure and was observed in F1 females may not be transgene-rationally inherited by females of the second generation. In fact, during critical developmental periods of fetuses exposed to environmental stimuli, the epigenetic phenotype becomes transgenerational once expressed in F2 [32,33]. Many environmental EDCs, such as bisphenol-A (BPA), di(2-ethylhexyl) phthalate (DEHP), and vinclozolin, have transgenerational effects [32]. The latter is a fungicide known to display transgenerational inheritance after ancestral exposure to this fungicide. Indeed, there was no increase in polycystic ovarian syndrome or primary ovarian insufficiency in F1 and F2 after in utero exposure to vinclozolin, but there was an increase in these two ovarian diseases in the third generation associated with changes in DNA methylation [30,34]. Thus, the mycotoxin FB1 disrupted the ovaries of F1 females in association with hypermethylation, but this disruption was not observed in F2 females. The absence of third-generation females to determine whether FB1 has a transgenerational impact represents one of the limitations of the current study.

Many studies have shown that epigenetic modifications alter different signaling pathways, leading to damage and cell death [35,11]. Among these signaling pathways, the Pi3k/Akt/mTORr signaling pathway has been reported to play a crucial role in governing various aspects of ovarian function. This pathway is involved in regulating the quiescence, activation, and survival of primordial follicles, as well as the proliferation and differentiation of granulosa cells and the meiotic maturation of oocytes [36,24]. Disruptions or abnormalities in these signaling pathways can have significant impacts on metabolic processes and the fate of ovarian cells, ultimately contributing to the development of infertility due to compromised follicular development, intrafollicular oocyte maturation, and ovulation [37,38]. Our findings demonstrated a significant increase in the expression levels of *Pi3k* exclusively in the group exposed to a high dose of FB1 in both the first and second generations compared to the control group (Fig. 4). This finding suggests that in utero exposure to a high dose of FB1 (50 mg/kg) has an intergenerational impact through *Pi3k*, which has the potential to disrupt ovarian function. It has been reported that excessive activation of the *Pi3k* signaling pathway results in premature activation of the entire pool of primordial follicles, ultimately leading to their depletion during early adulthood and the onset of premature ovarian failure [39]. This finding is consistent with the histopathological results, which showed an increase in the number of degenerating follicles, primarily in the group exposed to a high dose of FB1. Additionally, previous research has reported that a high dose of FB1 (50 mg/kg b.w.) decreases the number of primordial follicles, resulting in an expedited decline in reproductive capacity associated with aging [8].

Akt has been recognized for its significant role in a wide range of cellular processes, including the regulation of mammalian oocyte growth and the activation and early development of ovarian follicles [40]. This kinase collaborates with other kinases, including PI3K, to regulate coordinated follicle and oocyte development [39,40]. Our findings indicated that while there were no notable changes in the mRNA levels of the Akt gene in either F1 treatment group, a significant increase was observed in the second-generation animals whose parents were exposed to a high dose of FB1 (50 mg/kg) *in utero*. Since *Pi3k* was activated in the F2 animals whose parents were exposed to a high dose of FB1 (50 mg/kg b.w.) during fetal life, it may have activated Akt kinase in the females of the same group. The deletion of the protein PTEN in mouse ovaries, which is responsible for the maintenance of the pool of primordial follicles, has been reported to cause Akt hyperactivation, leading to rapid depletion of primordial follicles and early ovarian aging [39,41]. However, Akt upregulation could serve as a protective mechanism against the toxic effects of a high dose of FB1 on ovarian cells, as Akt can modulate apoptosis and regulate cell survival and proliferation [40]. Indeed, studies have shown that the overexpression of constitutively active Akt can effectively prevent cell death induced by neurotoxins and maintain the sprouting of axons in dopamine neurons located in the substantia nigra [42]. Consequently, Akt upregulation may play a role in the recovery mechanism, potentially working in conjunction with other factors such as autophagy and DNA methylation processes [8]. This finding aligns with the proposed therapeutic approach of targeting Akt for the treatment of various diseases [42].

Mammalian target of rapamycin (mTOR) is a serine/threonine kinase that plays a vital role in regulating cell growth and proliferation in response to various signals. Our findings indicate a significant decrease in the mRNA levels of the mTOR gene in both treatment groups of the first generation. However, there was no noticeable effect observed in the second-generation treatment groups. This suggests that if FB1 has a direct effect on *mTOR* gene expression in the ovaries of first-generation females during fetal development, this effect is not passed on to the second generation. Supporting this finding, a previous study demonstrated that exposure to FB1 during fetal development led to a decrease in the number of primordial follicles and reduced fertility rates in female rats of the first generation by impacting follicle growth and development [8]. Additionally, research has shown that inhibiting mTOR in granulosa cells disrupts follicle growth and causes mitotic abnormalities, while activating mTOR in granulosa cells promotes follicular development [43,44]. However, these effects were not inherited by the second generation, as no significant changes in the mRNA levels of the mTOR gene were detected. These changes in *mTOR* gene expression observed in the first generation and its regulation in the second generation may be linked to changes in the DNA methylation across both generations. Specifically, the downregulation of *mTOR* gene expression in both treatment groups of the first generation could be attributed to hypermethylation, as indicated by the increased expression of Dnmt3b. Conversely, the recovery observed in the second generation may be related to the modulation of Dnmt3b within this group. This hypothesis aligns with previous findings suggesting that injury in glucose-induced podocytes can be mitigated by inhibiting the *Pi3k/Akt/mTOR* signaling pathway in conjunction with DNA methylation changes [45]. However, further research is needed to clarify this relationship.

AMPK, a serine/threonine kinase, is highly conserved across eukaryotes and serves as a crucial cellular energy sensor. AMPK is activated when there is a shortage of ATP [46]. Additionally, AMPK can be activated in response to physiological or pathological conditions, as well as by certain drugs. Notably, AMPK activation is reduced in thecal cells associated with PCOS, but treatment with metformin increases AMPK activity in these cells. AMPK may play a role in reproductive processes, particularly in ovarian function, by regulating steroidogenesis in ovarian cells and promoting germ cell maturation. In primary rat granulosa cells, follicle-stimulating hormone (FSH) stimulates cell proliferation by upregulating cyclin D2 mRNA expression and inhibiting Ampk activation through an Akt-dependent pathway [47]. Our findings demonstrated that Ampk was not affected by FB1 exposure in either treatment group or in either generation.

## Conclusions

This study highlights the intergenerational effects of fetal exposure to fumonisin B1 (FB1) on ovarian function in Wistar rats, particularly through epigenetic modifications and alterations in key signaling pathways. Exposure to high doses of FB1 during fetal development induced global DNA hypermethylation in first-generation (F1) females, as evidenced by increased DNMT3b protein expression, which was associated with ovarian disruption. However, this effect was not observed in second-generation (F2) females, suggesting that the epigenetic changes induced by FB1 are not transgenerationally inherited. Additionally, FB1 exposure caused dose-dependent disruptions in the *Pi3k/Akt/mTOR* signaling pathway, with *Pi3k* overexpression observed in both F1 and F2 generations, while *Akt* and *mTOR* showed generation-specific alterations. Histopathological analysis confirmed ovarian damage, including degenerating follicles and abnormal oocyte structures, particularly in high-dose-treated groups. These findings demonstrate that FB1 exposure during early development can reprogram ovarian function *via* epigenetic and signaling pathway disruptions, potentially contributing to reduced fertility. However, the absence of transgenerational inheritance beyond the F1 generation underscores the complexity of epigenetic reprogramming. Future studies using advanced techniques such as single-cell sequencing and epigenome-wide analyses are warranted to further elucidate the molecular mechanisms underlying FB1-induced ovarian toxicity and its long-term reproductive implications.

## Figures and Tables

**Table 1:** Primers for real-time RT-PCR.

Gene Symbol	Sequences
*Pi3k*	F: GATGTCTGCGTTAGGGCTTACC
	R: TCAGCATCATGGGGAT
*Akt1*	F: CTCATTCCAGACCCACGAC
	R: ACAGCCCGAAGTCCGTTA
*mTOR*	F: TGCCTTCACAGATACCCAGTAC
	R: AGGTAGACCTTAAACTCGGAC
*Ampk*	F: ACCTCAGCGCTCCTGTTCTG
	R: TCTCGGCTGTGCTGGAATC
